# Relationship between workload and mind-wandering in simulated driving

**DOI:** 10.1371/journal.pone.0176962

**Published:** 2017-05-03

**Authors:** Yuyu Zhang, Takatsune Kumada

**Affiliations:** Department of Intelligence Science and Technology, Graduate School of Informatics, Kyoto University, Kyoto, Japan; Beihang University, CHINA

## Abstract

Mental workload and mind-wandering are highly related to driving safety. This study investigated the relationship between mental workload and mind-wandering while driving. Participants (N = 40) were asked to perform a car following task in driving simulator, and report whether they had experienced mind-wandering upon hearing a tone. After driving, participants reported their workload using the NASA-Task Load Index (TLX). Results revealed an interaction between workload and mind-wandering in two different perspectives. First, there was a negative correlation between workload and mind-wandering (r = -0.459, p < 0.01) for different individuals. Second, from temporal perspective workload and mind-wandering frequency increased significantly over task time and were positively correlated. Together, these findings contribute to understanding the roles of workload and mind-wandering in driving.

## Introduction

Driving a vehicle is a complex task that requires not only physical skills for controlling the direction and speed of a vehicle but also mental skills for sustained monitoring of integrated perceptual and cognitive inputs that allow a driver to make time-appropriate decisions [1]. Factors affecting a driver’s mental abilities in these contexts have been extensively investigated. Among the pertinent factors examined, mental workload, defined as the ratio of demand to allocated resources, has been identified as particularly critical to driving performance [2]. Especially, in driving a high mental workload appears to increase the risk of accidents.

Recently, the relationship between mind-wandering and driving has become the focus of research [3, 4]. Mind-wandering denotes a shift of attention away from the primary task, toward mentally internalized information [5]. Mind wandering is a common phenomenon during monotonous tasks, such as driving; where it is may impair a driver’s ability to respond to hazards. Accordingly, understanding the effects of mind wandering on driving is important to insuring driving safety.

The present study considers the relationship between mental workload and mind-wandering. Although both are considered to affect cognitive activity during driving, to the best of our knowledge, no attempt has made to examine this issue in detail. The load theory of attention [6] provides a useful theoretical background for integrating mental workload and mind-wandering. This theory assumes that individuals have a fixed capacity for cognitive control. Furthermore, it invites speculation that tasks involving a high perceptual load would engage all available capacity, leaving no additional capacity for perception of irrelevant distractors. In contrast, in the case of spare capacity, namely an individual’s residual capacity that extends beyond the needs for task-relevant processing of low perceptual load tasks, there is a potential for this capacity to involuntarily spill over into processing of irrelevant distracters. In turn, this leads to failures in selection of task-relevant information. In load theory irrelevant distractors have been extended to include internal sources of distraction caused by mind-wandering [7]. In other words, spare mental capacity may induce mind-wandering.

It is well established that situations with high mental workloads consume a great amount of mental capacity. De Waard (1996) [2] proposed a model of mental workload, task performance, and demands. In this model, when an operator can easily cope with task demands and reach a self-set adequate level of performance, the workload is low (referred to as Regions A2); when the operator has to exert effort to preserve performance, the workload increases (referred to as Regions A1 and A3). There are two kinds of effort to exert: task-related effort (effort for tasks that require controlled information processing) and state-related effort (the effort that an individual has to change the state of current energetic resources in the direction of resources of required state). An operator can maintain a performance level by increasing task-related effort (referred to as Regions A3). In addition, for instance, monotony starts to affect an operator’s internal state, i.e., with activity attenuation, and this tendency toward deactivation then is countered by state-related effort (referred to as Regions A1). In driving case, an experienced driver may devote less task-related effort and have lower workload compared with a novice driver attempting the same task. When a driver exerts a great amount of effort to counter a vigilance decrement (state-related effort) after a long period of driving, a state of high workload would appear.

Mental workload is defined as the ratio of task demand to allocated resources. Currently ‘task demand’ and ‘allocated resources’ cannot be measured directly. In other word, we cannot measure real-time mental workload directly. However, there are three categories of indirect measurements of mental workload, namely self-reported measures, performance measures (including primary-task and secondary-task performance measures), and physiological measures [2]. In the present study, we use self-reported measures and primary-task performance measures. Self-reported measures, such as the NASA-Task Load Index (TLX), assume that individuals subjectively perceive mental workload as a cost, and these measures summarize the influences of many factors, in addition to objective demands imposed by the task [8]. Since individuals commonly provide self-reported measures by responding to a set of questions only after a task is completed, data collected cannot reflect real time variations in mental workload during a task [9]. Primary-task performance measures are defined as measures of the overall effectiveness of man-machine interactions [2], such as standard deviation of lateral position (SDLP) and standard deviation of steering-wheel movements (SDSTW), which could reflect real time variations of mental workload in driving.

Mind-wandering is a mental state featuring limited external information processing in which attention is decoupled from the environment and oriented toward internal information [5]. It has been associated with lower levels of alertness and vigilance [10, 11]. Supporting this view, a few studies on mind-wandering in driving situations have reported that during mind-wandering participants have longer response times to sudden events, drive at higher speeds, maintain a shorter inter-vehicle separation distance [3], while tending to focus visual attention narrowly on the road ahead [4]. Moreover, Thomson et al. (2014) [12] suggested that the longer that attention is focused on a given external task, the more likely it is that mind-wandering will occur.

In this study, we used a thought sampling method to measure mind-wandering; this is the most common method used in mind-wandering studies. Mind-wandering by its very nature is a spontaneous, task-unrelated, internal mental process of which the subject is usually unaware. Consequently, it is typically difficult to study, document, or replicate mind-wandering using classical psychophysical paradigms [13]. Here we use a probe-caught mind-wandering method; during a driving task, participants are interrupted by a probe in the form of a brief tone. Next, they are asked to report their experiences related to mind-wandering just before the tone was given [5].

In the present study, the experiment was conducted in a driving simulator. The main task for participants was to follow a vehicle driving in front of them at a fixed distance, while also responding to tone probes to report mind-wandering state. After completing the driving task, participants filled a NASA-TLX form. We analyzed the relationship between mental workload and mind-wandering from two different perspectives.

As already noted, the load theory of attention predicts that spare capacity induces mind-wandering. For different individuals, the difficulty of task used in this study is different and there would be have different spared capacity. It is reasonable to predict that mental workload, which is a subjective measure of load to capacity (referred to as ‘Workload-I’), negatively correlates with mind-wandering (referred to as ‘Mind-wandering-I’) (where ‘I’ refers to ‘individual’) for different individuals. Since this subjective measure of mental workload is only available after a task, we compared it to the total number of mind-wandering reports during the task.

Another relationship between mental workload and mind-wandering addressed in this study is from temporal perspective. As participants drove on a simple road in a simulator, their state gradually changed from region A2 to region A3 in the De Waard (1996) [2]’s model. After a long period of driving, decreasing concentration to external events may cause perceptual decoupling and increased mind-wandering. In order to counteract mind-wandering, drivers must exert a great amount of effort to avoid vigilance decrement (termed here state-related effort). This leads to an increased awareness of mental workload. This situation leads us to predict a positive correlation between workload and mind-wandering frequency over time in the task. We refer the performance measure of mental workload over this time course as ‘Workload-T’ and the measure of mind-wandering frequency as ‘Mind-wandering-T’ (where ‘T’ refers to ‘temporal’).

## Methods

### Participants

This study was approved by the Ethics Committee in Unit for Advanced Studies of the Human Mind, Kyoto University. Participants were recruited from Kyoto University (N = 40, Age range 20–39 years, Mean age 22.475 years, SD = 2.987) and volunteered for this experiment after providing written informed consent. All participants held a valid Japanese driver’s license. They received 2000 yen (about 20 USD) for their participation in the study.

### Apparatus and stimuli

**Fig 1:**
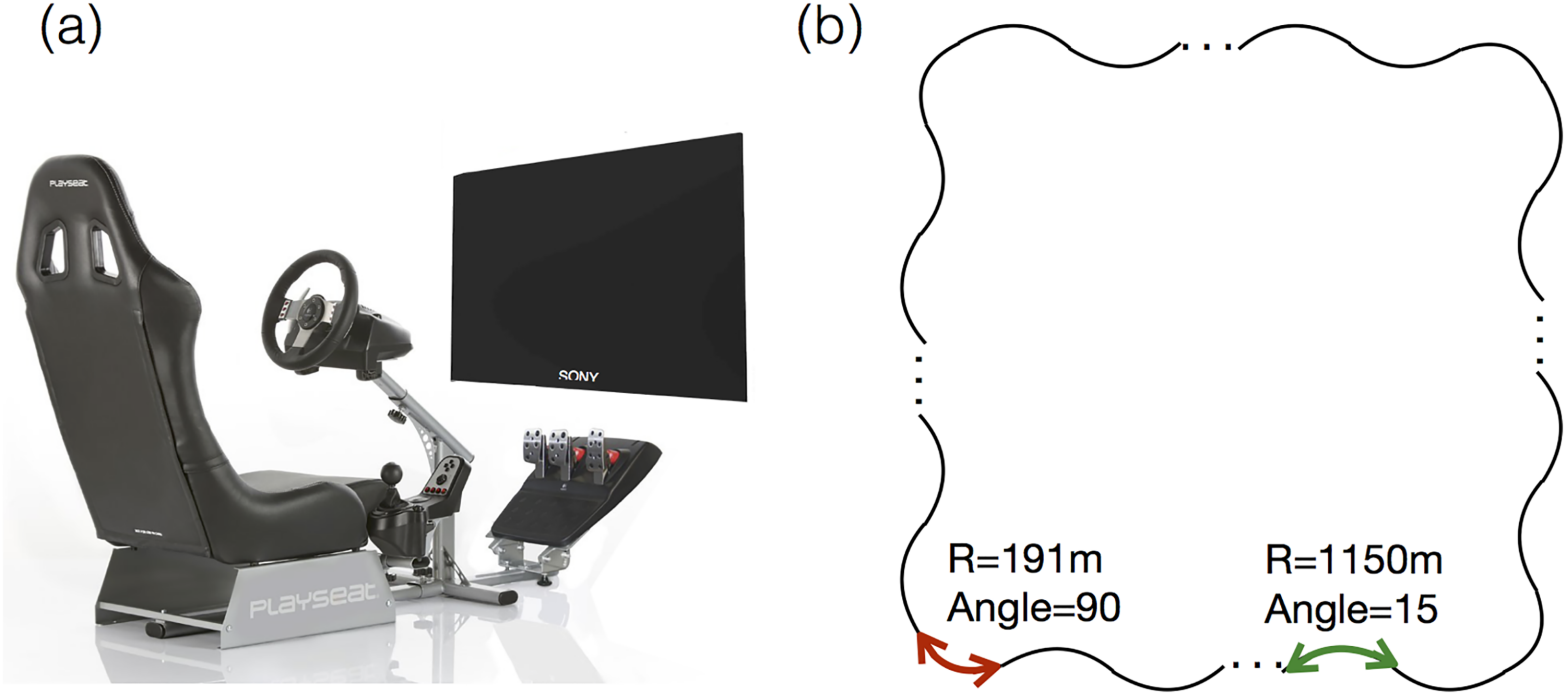
Overview of the driving simulator (a) and track design (b).

The experiment was conducted in a driving stimulator (Fig 1a, Play-seat Evolution Black + Logitech G27) equipped with all devices needed to operate a vehicle, including a steering wheel, accelerator, brake pedals, and a gearshift. Buttons on the steering wheel allowed participants to react to sounds. The stimulator had a display (SONY FWD-S42H2), featuring a very high picture quality (1920 pixels *1080 pixels) with full high definition and high brightness. Driving environments and traffic scenarios were created with a simulator software (UC-win/Road 10.4, FORUM8 Inc.) and displayed on the screen. Driving performance was measured at sampling rate of 10Hz.

The driving environment simulated good weather conditions in daylight; the driving track is shown in Fig 1b. The track had two lanes and the width of each lane was 3.2 meters. The length of track is 25, 270 meters.

### Procedure

In an experimental session, participants were required to follow a lead vehicle by maintaining a distance of 20 m without deviating from the lane. The lead vehicle was programmed to maintain a speed of 80km/h, with the exception of braking at randomly selected times. When the lead vehicle braked, participants also had to brake to avoid a rear-end crash.

While participants performed the task of car-following, there were asked to also respond to brief thought probes tone (last 0.3s). After hearing a tone while driving, participants were instructed to report whether they were mind-wandering based on their thought content immediately preceding a tone’s occurrence. If they had been mind-wandering, they pressed the red button on the steering wheel close to their right hand; if they had not been mind-wandering, they pressed the red button on the steering wheel close to their left hand. Mind-wandering was defined to participants as having thoughts unrelated to driving task, such as planning a schedule, or as having a blank mind. Thoughts about maintaining the appropriate distance from the lead vehicle, braking and accelerating, and staying in the lane were not mind-wandering.

A driving task lasted for 25 min. With the exception of the first minute, every minute the lead vehicle’s braking and the tone probe appeared once. There were 24 probes and 24 braking in this driving task. The tone probe did not appear during braking and during turning direction (the corner of the square) on the 5th, 10th, 15th, 20th and 25th min. Driving performance data in first minute was not included to analyze.

After driving, participants were instructed to complete the NASA-TLX report. This report comprises six sub-scales: mental demand, physical demand, temporal demand, overall performance, frustration, and effort. Each scale has a point range with 5-point steps and is anchored by bipolar descriptors.

Before an experimental session, two practice sessions were given to participants. In the first practice session, participants learned to drive keeping a 20 m distance from the lead vehicle. When the distance between a participant’s vehicle and a lead vehicle was not between 17 m to 23 m, the messages ‘far’ or ‘close’, appeared on the screen to remind participants to adjust their distance. The driving task ended when participants could maintain the appropriate distance for 1 minutes. In the second practice session, participants completed the car following task with mind-wandering reporting and NASA-TLX report, both of which were the same as in the experimental session. This practice continued for 5 min.

## Results

### Workload-I and Mind-wandering-I

**Fig 2:**
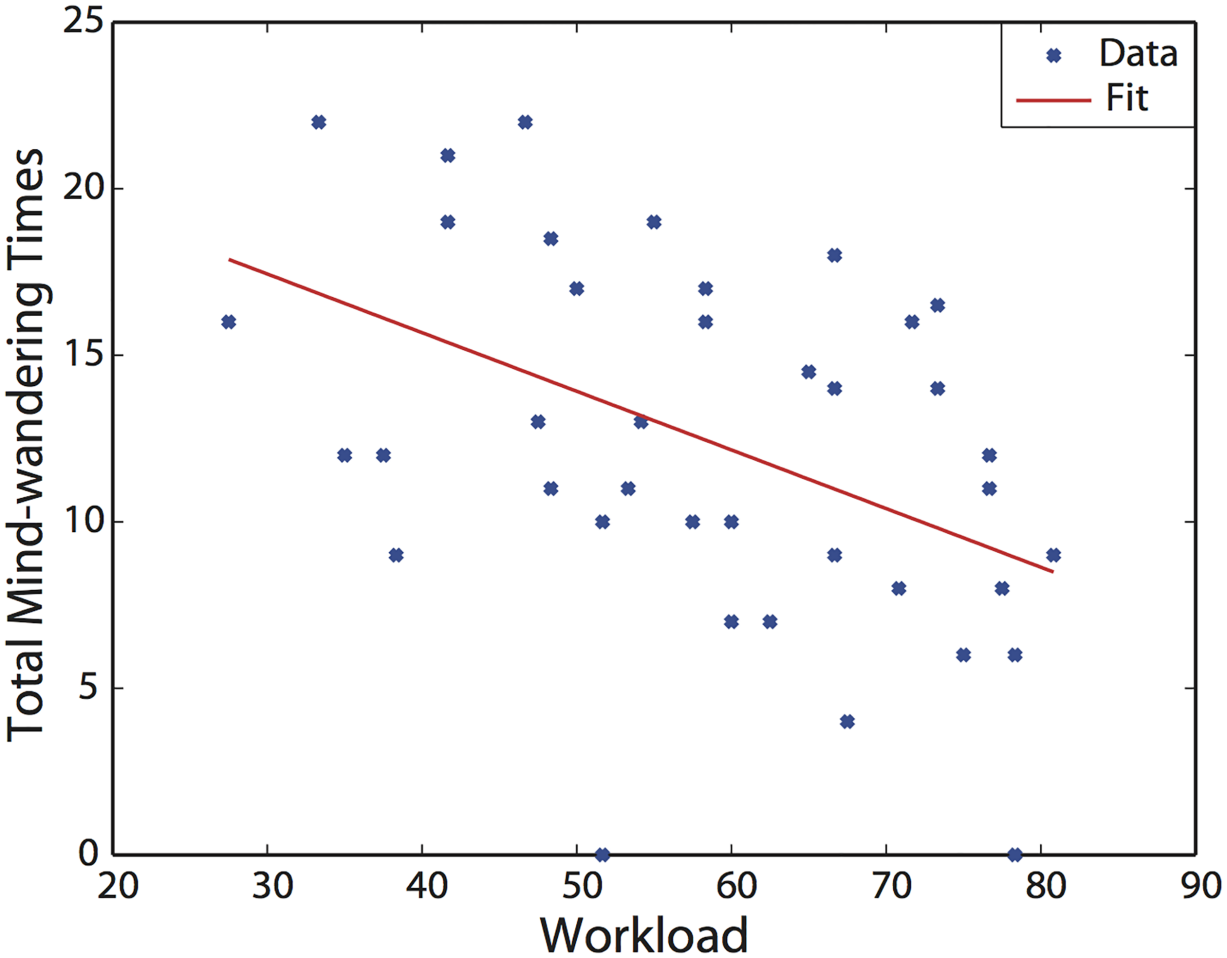
Linear regression model of Workload-I and Mind-wandering-I.

Workload-I is indicated by the workload reported with NASA-TLX after driving; it was calculated by averaging the six sub-scales of NASA- TLX is referred to as the Raw TLX (RTLX) [14]. Mind-wandering-I is indicated by total number of mind-wandering incidents reported by participants after probes. Fig 2 shows scatter plot of workload values as a function of mind-wandering reports. The Pearson’s linear correlation coefficient was statistically significant (r = -0.459, p < 0.01); this was also the case for the linear regression model (p < 0.01, R-squared = 0.21).

### Workload-T and Mind-wandering-T

The Workload-T during driving process is indicated by the primary-task performance measures. The main driving tasks here were keeping the car in the lane and maintaining a certain distance from the leading vehicle. Therefore we use standard deviation of lane position (SDLP) and standard deviation of steering wheel movements (SDSTW) to describe lateral control performance; we also use standard deviation of foot operation (SDFO) and standard deviation of distance (SDD) to describe longitudinal control performance. SDLP was analyzed as the standard deviation of vehicle offset from the center of the lane in meters, and SDSTW was analyzed as the standard deviation of steering wheel ratio, such that a maximum left turn was -1, and a maximum right turn was 1. The SDFO indicated the extent of variability of foot movements, including pressing the accelerator or brake pedal. If the accelerator pedal was pressed and the subjects’ car was accelerating, the operation was positive; if the brake pedal was pressed and subjects’ car was decelerating, the operation was negative. The minimum and maximum values of SDFO were -1 and 1 respectively. The SDD indicated the variability of distance between a participants’ car and the front car. Here we calculated these performance measures at every minute, then averaged data for all participants in corresponding minutes of each measure and conducted Mann-Kendall trend testing.

**Fig 3:**
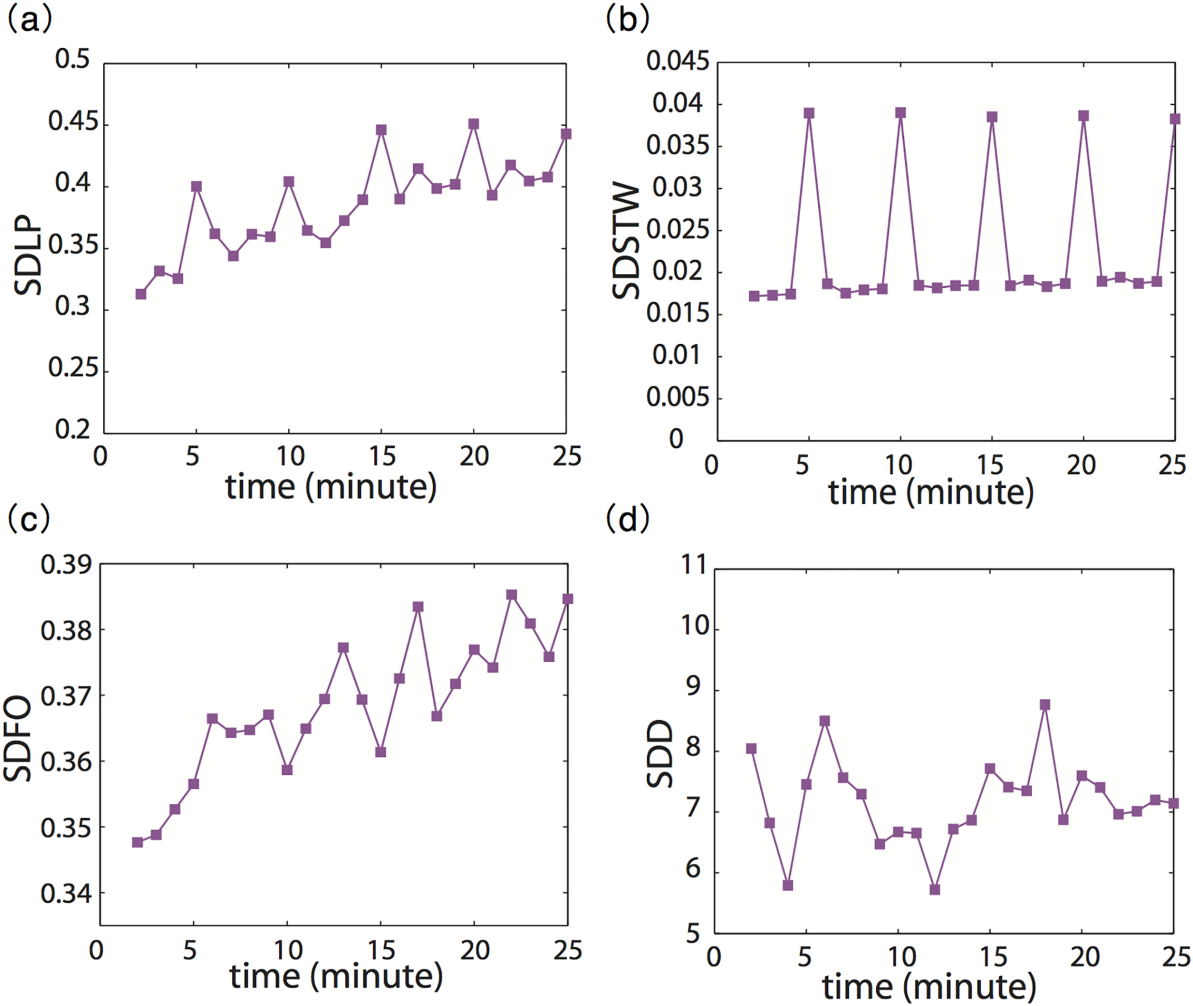
Performance measures with progress of time (Workload-T). The SDLP and SDSTW were autocorrelated, but this was not the case for SDFO and SDD. The SDLP, SDSTW and SDFO significantly increased with time (p < 0.01, sen > 0), but this did not happen for SDD.

The nonparametric Mann-Kendall test based on the assumption of independent observations is generally used to detect an increasing or decreasing trend in a time series [15]. In the trend testing, we took the effect of serial correlation of the time series into account [16]. Residual autocorrelation was plotted along with the approximate 95%-confidence intervals [17]. The time series was auto-correlated to determine if autocorrelation value had more than 5% chance of being outside the 95%-confidence intervals, except for a lag 0 [18]. If the time series was not autocorrelated, a Mann-Kendall test was directly performed; if the time series was autocorrelated, a modified Mann-Kendall test with variance correction (MKDD) was performed [19]. Also calculated the Sen slope as an estimate of this trend [20, 21]. Fig 3 shows tests for these measures as a function of time. Results indicated that SDLP and SDSTW were autocorrelated, but SDFO and SDD were not. All performance measures significantly increased with time (p < 0.01, sen slope > 0), except for SDD (p > 0.05).

**Fig 4:**
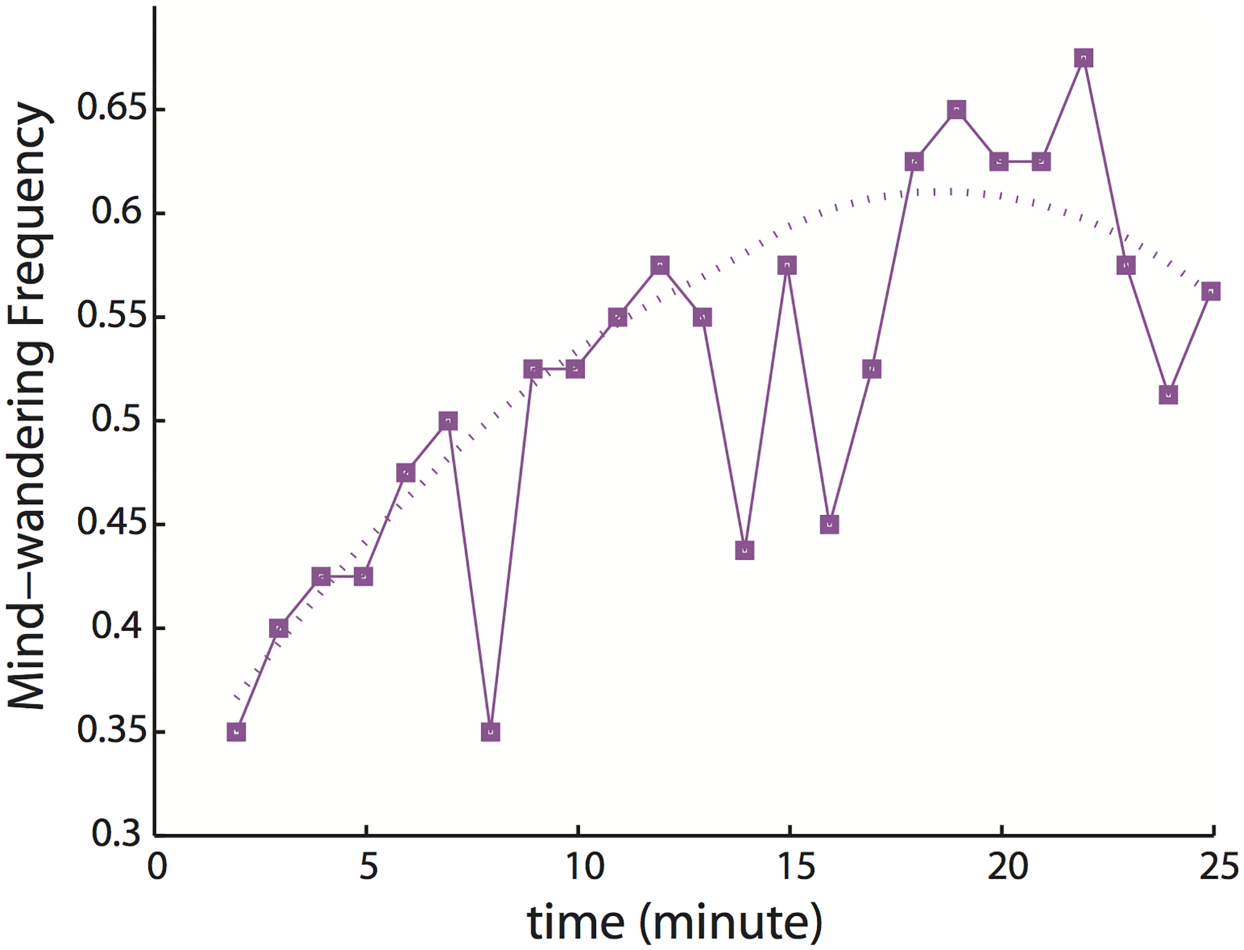
Mind-wandering frequency with progress of time (Mind-wandering-T).

Mind-wandering-T was indexed by the frequency of mind-wandering during driving. Mind-wandering was assigned a value of 1 if participants reported mind-wandering, and a value of 0 if participants reported no mind-wandering; a value of 0.5 indicated that a participant reported nothing (only 3 occasions for all participants). The mean of mind-wandering values of all participants in corresponding minute was defined as mind-wandering frequency. We next conducted trend tests on it. Fig 4 shows mind-wandering frequency (Mind-wandering-T) as a function of time. The time series of mind wandering frequency was not autocorrelated and the trend showed significant increases over time (p < 0.01, sen slope > 0). To emphasize the shape of the relationship between mind-wandering frequency and time, we conducted robust LOESS smoothing (derived from the term “locally weighted scatter plot smooth”) with a span of 0.9. The smoothed curve was plotted as a dotted line in Fig 4.

**Fig 5:**
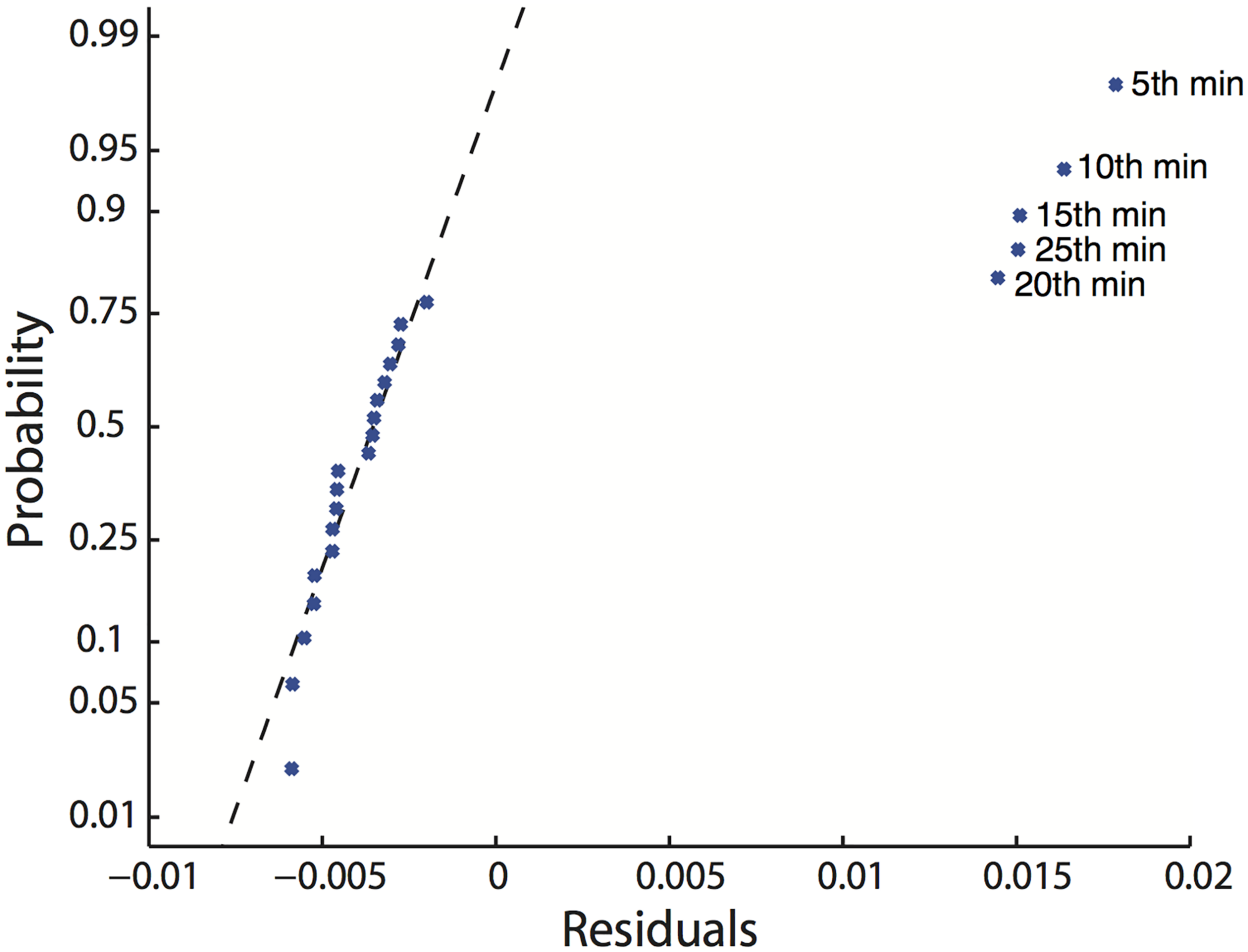
Probability plot of the residual of the linear regression model of SDSTW and mind-wandering frequency.

We performed a Pearson’s correlation coefficient calculation and linear regression analysis for each performance measure and mind-wandering frequency. Table 1 summarizes the analysis. The correlation coefficient of SDLP and mind-wandering frequency was significant (r = 0.622, p < 0.01) and linear regression model was fit (R-squared = 0.387), so were SDFO and mind-wandering frequency (r = 0.650, p < 0.01, R-squared = 0.422). Fig 5 shows the probability plot of the residual of the linear regression model of SDSTW and mind-wandering frequency. As for SDSTW and mind-wandering frequency, the residual was not normally distributed; the data points in the 5th, 10th, 15th, 20th and 25th minutes were too far from the diagonal reference line, and were considered outliers [16]. After removing outliers, the correlation coefficient of SDSTW and mind-wandering frequency was significant (r = 0.695, p < 0.01) and a linear regression model was built (R-squared = 0.482). The residual of the linear regression model of SDD and mind-wandering frequency showed normal distribution, but the correlation coefficient was not significant.

**Table 1:** The Pearson’s correlation coefficient and linear regression model of each performance measures and mind-wandering frequency.

	Is normal distribution	Correlation coefficient	Linear regression model (y = b+a*x)		
			a	b	R-squared
SDLP	Yes	0.622 (p < 0.01)	0.254 (t = 3.727, p < 0.01)	0.253 (t = 7.026, p < 0.01)	0.387
SDSTW	No	(p > 0.05)	-	-	-
SDSTW (remove outliers)	Yes	0.695 (p < 0.01)	0.005 (t = 3.981, p < 0.01)	0.016 (t = 26.883, p < 0.01)	0.482
SDFO	Yes	0.650 (p < 0.01)	0.076 (t = 4.011, p < 0.01)	0.329 (t = 32.958, p < 0.01)	0.422
SDD	Yes	(p > 0.05)	-	-	-

## Discussion

In this study, we examined the relationship between mind-wandering and mental workload in a simple car-driving task. This relationship was mainly analyzed from two perspectives: individual difference perspective and time perspective. In the following section, we discuss the result of each perspective.

With respect to individual differences, we found a negative correlation between mind-wandering and mental workload. Specifically, individual drivers who experienced a higher workload reported fewer instances of mind wandering (see Fig 2). This is consistent with a prediction based on the load theory of attention. Participants who register a lower mental workload will have spare capacity; in turn, this spare capacity is allocated to the internal task-irrelevant information, leading to mind-wandering. This is also consistent with recent understanding of attentional control. It is known that attentional resources for internally directed cognition (IDC), which involve internally attending to thoughts, memories and mental imagery, and externally directed cognition (ECD), which involves attending to stimuli in the external environment, are not conducted in an all-or-none fashion. Instead, these resources can be divided into any proportional relationship to achieve a dynamic balance [22]. This enables participants to drive despite the presence of on-going mind-wandering. It is also known that IDC depends on top-down regulatory processes to ensure that relevant goals are achieved, suggesting that processing capacity is primarily allocated to internal goals for driving, not to mind-wandering [23]. Consequently, participants who allocate larger capacity for EDC or IDC to driving goals will have less capacity for mind-wandering, as the total process capacity is limited. Thus, these participants may report higher mental workload and experience fewer instances of mind-wandering.

Compared to the present study, some previous studies of mental workload during driving tend to use more complex tasks in an attempt to mirror the complexity of real world tasks and environments [24]. For example, Zhang et al. (2004) [25] used two secondary tasks (verbal and spatial-imagery) and Kawakita et al. (2010) [26] used three secondary tasks (listening to music, conversation, and arithmetic) during driving. Here we measured participants’ driving behavior in a simple monotonous situation, in which no other traffic or secondary driving distraction task appeared. The present driving task merely required participants to control lateral and longitudinal positions. Therefore, we considered that the driving measures adopted in this study should be relatively free from situational factors caused by a complex environment. This interpretation is also supported by the fact that primary-task performance measures revealed a significant result in Fig 3, suggesting that primary-task performance measures are sensitive and sufficient to indicate workload level variation in this task.

In this study, we used four driving performance measures, SDLP, SDSTW, SDFO and SDD, some of which have been commonly used as workload performance measures in previous studies. As shown in Fig 3, these performance measures mainly demonstrated significantly increasing trends over time (except for SDD), showing that driving performance worsens as a function of time. Degraded driving performance could indicate an increase of mental workload over time. With respect to the longitudinal control measure, however, there was a significant trend for SDFO, but not for SDD, possibly because it is difficult for participants to accurately know how far 20 meters is on the stimulator screen even though they had been trained to keep this distance.

Mind-wandering frequency (Mind-wandering-T) significantly increased with time (see Fig 4). Similar results have been demonstrated in several other studies [12, 27]. The smoothed mind-wandering frequency line started with 0.3-0.4, which is consistent with the 30%—40% frequency of mind-wandering in daily life [28]. After about 17 to 24 min, mind-wandering frequency trended to decrease and therefore likely to have a ceiling, which may be due to the limited processing capacity. The performance measures, SDLP, SDSTW and SDFO, were positively correlated with mind-wandering frequency, suggesting that generally mental workload has a positive correlation with mind-wandering.

In discussing the time-dependent increasing trend of mental workload and mind-wandering, we should consider the effect of fatigue over time. It is known that workload and fatigue can increase simultaneously [29, 30]. The level of workload generally increases as a function of the length of work period [31, 32]. Mental fatigue also gradually cumulates with additional work time [33]. Although mental fatigue decreases the capability of conducting physical or mental work [34], for the present context, it may be important to consider the fact that, under fatigue, executive control is compromised and consequently causes deficits in task performance [35, 36]. The executive control ability to keep one’s goals (and goal-relevant representations) mentally active and accessible is fundamental to task performance [37]. Engagement of executive control can also prevent mind-wandering. When executive-control is deficient, mind-wandering is more likely [38, 39]. As a result, an increase in mental fatigue with time can result in decreased executive control capability and an increase in spontaneous mind-wandering. Apparently, further research is required to clarify the relationships among mental workload, fatigue, and mind-wandering.

The result that both Workload-T and Mind-wandering-T increased over time may be explained from another perspective. Mind-wandering is considered to be useful as it provides mental breaks to alleviate boredom from monotonous activities [40]. When mental workload increases with time, our brain self-regulates to increase spontaneous mind-wandering in order to ease the brain’s workload.

In the present study, we have demonstrated the interaction between workload and mind-wandering from two different dimensions: one from the individual difference dimension in which participants with higher workload have fewer instances of mind-wandering; the other from the temporal dimension in which both workload and mind-wandering increase with task time. Although results from simple laboratory situations used in this study may not generalize directly to practical and complex situations, these results can refine theories about workload and mind-wandering to solve practical problems. As a practical issue, the high correlation between workload and mind-wandering suggests the possibility that mind-wandering may be a reliable indicator of workload. It may be difficult to ask about subjective mental workload for drivers. However, we suggest that drivers’ workload might be estimated by questioning them about mind-wandering status during driving. This type of reporting may be relatively easy for drivers and should be further investigated in the future.

## Supporting information

S1 Dataset Data used to plot Figs 2, 3, 4 and 5. (XLSX)
